# Metabolic Responses of a Model Green Microalga *Euglena gracilis* to Different Environmental Stresses

**DOI:** 10.3389/fbioe.2021.662655

**Published:** 2021-07-20

**Authors:** Jiayi He, ChenChen Liu, Mengzhe Du, Xiyi Zhou, Zhangli Hu, Anping Lei, Jiangxin Wang

**Affiliations:** ^1^Shenzhen Key Laboratory of Marine Bioresources and Eco-environmental Science, Shenzhen Engineering Laboratory for Marine Algal Biotechnology, Guangdong Provincial Key Laboratory for Plant Epigenetics, College of Life Sciences and Oceanography, Shenzhen University, Shenzhen, China; ^2^Key Laboratory of Optoelectronic Devices and Systems of Ministry of Education and Guangdong Province, College of Physics and Optoelectronic Engineering, Shenzhen University, Shenzhen, China; ^3^College of Chemistry and Environmental Engineering, Shenzhen University, Shenzhen, China

**Keywords:** *Euglena*, metabolomics, antibiotics, heavy metals, nutrient deprivation, environmental stresses

## Abstract

*Euglena gracilis*, a green microalga known as a potential candidate for jet fuel producers and new functional food resources, is highly tolerant to antibiotics, heavy metals, and other environmental stresses. Its cells contain many high-value products, including vitamins, amino acids, pigments, unsaturated fatty acids, and carbohydrate paramylon as metabolites, which change contents in response to various extracellular environments. However, mechanism insights into the cellular metabolic response of *Euglena* to different toxic chemicals and adverse environmental stresses were very limited. We extensively investigated the changes of cell biomass, pigments, lipids, and paramylon of *E. gracilis* under several environmental stresses, such as heavy metal CdCl_2_, antibiotics paromomycin, and nutrient deprivation. In addition, global metabolomics by Ultra-high-performance liquid chromatography tandem mass spectrometry (UHPLC–MS/MS) was applied to study other metabolites and potential regulatory mechanisms behind the differential accumulation of major high-valued metabolites. This study collects a comprehensive update on the biology of *E. gracilis* for various metabolic responses to stress conditions, and it will be of great value for *Euglena* cultivation and high-value [154mm][10mm]Q7metabolite production.

## Introduction

*Euglena gracilis*, which began to appear 500 million years ago, is a species of unicellular organisms that live mostly in freshwater (Goto and Beneragama, 2010) and has no cell walls, giving it dual characteristics of plants and animals, which enable this microalga to adopt photosynthetic, heterotrophic, and mixotrophic conditions during long-term evolution (Edmunds, 1965; Zakryś et al., 2017). During this long evolution, *Euglena* became a highly adaptable microorganism that survived diverse and extreme conditions on the earth, such as high UV radiation, acid mine water, man-made antibiotics, heavy metal pollution, and nutrient deprivation (Ferreira et al., 2007; Moreno-Sánchez et al., 2017).

Among microalgae, *E. gracilis* is well-known as a producer of polyunsaturated fatty acids (PUFAs) (Schwarzhans et al., 2015), vitamin E (Takeyama et al., 2015), chlorophyll a and b, several types of carotenoid pigments (Tanno et al., 2020), and polysaccharose paramylon (β-1,3-glucan) (Ivusic and Santek, 2015). Nitrogen deficiency (as nitrogen deprivation, N–) is one of the most common environmental stresses to enhance lipid (biofuels) accumulation in microalgae (Peccia et al., 2013). An increase of total lipids in stationary growth phase cells in comparison to exponential growth phase cells in N deprived, mixotrophic *E. gracilis* cultures had been reported (Regnault et al., 1990). And a decrease in chlorophyll production by *E. gracilis* was observed during a short-term exposure to N– (García-Ferris et al., 1996). A long-term N limitation on lipid, protein, and pigment production of *E. gracilis* in photoheterotrophic cultures indicated that N– could increase total fatty acid production, with lower protein contents and pigment production (Tossavainen et al., 2019). The symptoms of N deficiency are well-documented, but the underlying molecular mechanisms are largely unknown in microalgae (Tossavainen et al., 2019).

*Euglena gracilis* is a highly promising group of microorganisms to be used in the bio-remediation of heavy metal-polluted aerobic and anaerobic acidic aquatic environments. Considering the current data and the fact that *E. gracilis* has an innate tolerance to relatively high concentrations of heavy metals, as demonstrated by the minimum inhibitory concentrations (MICs) obtained, that is, CdCl_2_ (50 μM) induced diminution in cell growth (Castro-Guerrero et al., 2008) this organism has the potential for the management of particular heavy metal contamination of the environment (Khatiwada et al., 2020). In *E. gracilis*, CdCl_2_ exposure can induce morphological alteration, linked to reactive oxygen stress (Watanabe and Suzuki, 2002). So far, the connection between Cd, paramylon, and lipid accumulation in *E. gracilis* is still unclear.

*Euglena gracilis* was reported to be highly tolerant to various antibiotics (Shao et al., 2018). When bacteria and other microalgal cells were significantly inhibited by antibiotics at the low concentration of 20–50 μg/ml (50 μg/ml Kanamycin *Chlamydomonas*; Bateman and Purton, 2000), *E. gracilis* cells still grew well. In *Euglena*, rapamycin induced the reduction of chlorophyll and the accumulation of neutral lipids without deterring its cell proliferation. In another green microalga of *Chlamydomonas*, however, rapamycin induced serious growth inhibition as reported elsewhere (Mukaida et al., 2016). Various antibiotics were used to bleach *Euglena* with concentrations as high as 200 μg/ml (Ebringer, 1964). Based on our preliminary experiments, paromomycin (PRM) could not inhibit *E. gracilis* cell growth at concentrations ranging from 10 to 50 μg/ml. Since streptomycin significantly bleached *Euglena* cells by deleting part of the chloroplast genome, we did not choose streptomycin for further investigation in this study. Currently, no report of PRM against *E. gracilis* has been listed, and the antibiotic resistance mechanism of *E. gracilis* is still unclear.

The research on different high-value metabolites and their potential control mechanisms under various environmental stresses are especially important and interesting. However, because of the absence of a high-dimension genome of *E. gracilis* (Ebenezer et al., 2017) and with limited previous OMICS studies, there is no definite conclusion on molecular mechanisms involved in responses to different environmental stresses.

With the advent of the post-genome era, various OMICS technologies, such as proteomics and metabolomics, appeared one after the other, in which metabolomics was the first to develop and the most widely applied technology (Johnson et al., 2016). Metabolomics has been an important branch of system biology in recent years. It can help to understand the biological process more directly and effectively, and the study of metabolites can also help to analyze complex sample traits (Johnson et al., 2016). We used the metabolomic approach to investigate the effects of different inoculum sizes on a green microalga *Chlorella* growth (Lu et al., 2012), salt stress in a model cyanobacterium (Wang et al., 2014), astaxanthin induction in *Haematococcus* (Su et al., 2014), antioxidant butylated hydroxyanisole on lipid accumulation in *Crypthecodinium cohnii* (Sui et al., 2014), and obtaining better understanding on the metabolic response mechanism of microalgae with different environments.

In this study, we explored global metabolomics to analyze metabolite changes under various conditions in *E. gracilis* for the first time, that is, heavy metals, antibiotics, and nutrient deficiency, to explore the mechanism of tolerance, acclimatization, and high-value product accumulations. The results show that pigments, lipids, and carbohydrates (paramylon) can be altered significantly under different stresses. Further, comparative metabolomics revealed some common and unique metabolic pathways and modules regulated by all selected stresses and individual stress, respectively.

## Materials and Methods

### Strains and Culture Conditions

*Euglena gracilis* CCAP 224/5Z was obtained from the Culture Collection of Algae and Protozoa (https://www.ccap.ac.uk/). The microalgal cells were grown in the EM medium [1.8 g/L NH_4_Cl, 0.6 g/L KH_2_PO_4_, 0.6 g/L MgSO_4_, 60 mg/L urea, 0.02 g/L CaCl_2_, 0.48 mg/L Na_2_EDTA, 2 mg/L Fe_2_(SO_4_)_3_, 60 μl HCl, 0.01 mg/L Vb_1_, 0.0005 mg/L Vb_12_, 20 mg/L CuSO_4_·5H_2_O, 0.4 g/L ZnSO_4_·7H_2_O, 1.3 g/L Co(NH_3_)·H_2_O, and 1.6 g/L MnCl_2_·4H_2_O] in 6 ml culture volumes of a 6-well plate under a light intensity of ~100 μmol/m^2^/s in an illuminating incubator without shaking at 26°C until microalgal cells reached the stationary phase (Afiukwa and Ogbonna, 2007; Yanming et al., 2018).

### Cultivation and Stress Treatments

*Euglena gracilis* cells were cultured for 6 days in the EM medium (10 ml EtOH in 100 ml EM medium), then 1 × 10^6^ cells/ml were centrifuged at 5,000 × *g* for 3 min and transferred into an equal volume of the EM medium as the 1:10 dilution was applied to each treatment well in a 12-well plate. Treatments were applied, including supplementation with PRM (25 μg/ml), CdCl_2_ (Cd) (0.5 mM), or nitrogen deprivation (N–) (without NH_4_Cl nor urea). After 6 days, stationary phase cell samples were collected and used for future experiments.

### Cell Growth, Chlorophyll, Paramylon, and Total Lipids

Cell growth was measured by counting cell numbers with a microscope in a 0.1-ml counting chamber. The total algal chlorophyll was extracted with 95% ethanol, and the content was spectrophotometrically assayed according to the method (Harris, 2009; Smith, 2009). The paramylon of 5 × 10^7^-10^8^ cells was extracted and measured as described previously (Sugiyama et al., 2009; Nakazawa et al., 2017; Guo et al., 2020). The total lipids of 10^7^-10^8^ cells were measured using the oven-drying method (Yang et al., 2018).

### UHPLC–MS/MS Metabolite Analysis

For the LC–MS/MS analysis, samples took after 6 days of incubation were first centrifuged at 5,000 × *g* for 5 min at 4°C to harvest ~10^6^-10^7^ cells. Metabolite extraction was followed as per previously reported protocol (Zhang et al., 2020). Cell pellets were then transported to a 1.5-ml Eppendorf microcentrifuge tube (Hamburg, Germany). After adding 1,000 μl extract solvent (acetonitrile:methanol:water, 2:2:1, containing internal standard), the samples were vortexed for 30 s using a XW-80A vortex mixer (Kylin-Bell Lab Instruments Co., Ltd., Haimen, China), homogenized at 45 Hz for 4 min using a tissue grinding machine (JXFSTPRP-24, Shanghai Jingxin Industrial Development Co., Ltd., Shanghai, China), and sonicated for 5 min in an ice-water bath using an ultrasonic cell-crushing device (Fangao Microelectronics Co., Ltd., Shenzhen, China). The homogenate and sonicate circle was repeated three times, followed by incubation at −20°C for 1 h and centrifugation at 12,000 rpm 4°C for 15 min. The LC–MS/MS analyses were performed using a ultra-high-performance liquid chromatography (UHPLC) system (1290, Agilent Technologies, Waldbronn, Germany) with a UPLC HSS T3 column (2.1 mm × 100 mm, 1.8 μm) coupled to Q Exactive mass spectrometer (Orbitrap MS, Thermo Fisher Scientific, San Jose, CA, USA). Mobile phase A was 0.1% formic acid in water for positive mode, and 5 mmol/L ammonium acetate in water for negative mode, and the mobile phase B was acetonitrile. The elution gradient was set as follows: 0–1.0 min, 1% B; 1.0–8.0 min, 1–99% B; 8.0–10.0 min, 99% B; 10.0–10.1 min, 99–1% B; 10.1–12 min, 1% B. The flow rate was 0.5 ml/min. The injected volume was 2 μl. QE mass spectrometer was used for its ability to acquire MS/MS spectra information-dependent acquisition (IDA) mode in the control of the acquisition software (Xcalibur 4.0.27, Thermo Fisher Scientific, San Jose, CA, USA). In this mode, the acquisition software continuously evaluates the full-scan MS spectrum. The electrospray ionization (ESI) source conditions were set as following: sheath gas flow rate as 45 arb, Aux gas flow rate as 15 arb, capillary temperature 400°C, full MS resolution as 70,000, MS/MS resolution as 17,500, collision energy as 20/40/60 eV in normalized collision energy (NCE) mode and spray voltage as 4.0 kV (positive) or −3.6 kV (negative), respectively (Periannan, 2003).

Compound identification from a non-targeted metabolite database were converted to the mzXML format using ProteoWizard and processed with an in-house program, which was developed using R and based on XCMS, for peak detection, extraction, alignment, and integration. Then an in-house MS2 database (BiotreeDB) was applied in metabolite annotation. The cutoff for annotation was set at 0.3. (Smith et al., 2006). Identified peaks were normalized to the peak intensity of ribitol. The pathway enrichment analysis on the metabolite dataset based on Kyoto Encyclopedia of Genes and Genomes (KEGG) was performed in Metaboanalyst 3.0^1^ (Xia et al., 2015). Related assistance was performed at Shanghai Biotree Biotech Co., Ltd. (Shanghai, China).

### Statistical Analysis

Significant differences in growth, chlorophyll content, biomass, and paramylon content were tested using the Dunnett's *t*-test. All data were obtained and averaged from at least three independent experiments, and standard errors were calculated and displayed as error bars. And after raw data profiles were processed, the Student's *t*-test was used for the univariate analysis. Principal component analysis (PCA) and orthogonal projections to latent structures discriminant analysis (OPLS-DA) were used for multivariate analysis. Variable importance in projection (VIP) score was combined with *p*-value to screen significant differential metabolites, and then the significant differential metabolites were qualitatively analyzed based on the relevant KEGG pathway and existing materials (Mangalam et al., 2013).

## Results

### Cell Growth, Chlorophyll, Paramylon, and Lipids

Cell growth differed greatly under different environmental stresses (Table 1). The PRM treatment caused a slight decrease (7.17%, *p* > 0.05), whereas CdCl_2_ (Cd) and nitrogen deprivation (N–) treatments significantly inhibited the growth of *E. gracilis* by 7.79% (*p* < 0.05) and 11.95% (*p* < 0.05), respectively.

**Table 1:** Physiological parameter, cell density, chlorophyll contents, paramylon, and total lipids determined under different environmental stresses.

	**Cell density (10^**6**^ cell/ml)**	**Chlorophyll contents (mg/L)**	**Paramylon (μg/mg)**	**Total lipids (μg/mg)**
EM	1.037, 0.038	35.764, 1.495	53.073, 1.986	60.49, 7.713
PRM	0.963, 0.040	35.525, 1.541	42.187, 7.721	70.099, 1.201
N–	0.913, 0.044^*^	22.543, 1.406^**^	35.133, 0.590^*^	138.732, 3.442^**^
Cd	0.956, 0.025^*^	16.417, 0.356^*^	84.310, 4.560^*^	92.704, 8.928^*^

*EM, control; PRM, paromomycin; N–, nitrogen deprivation; Cd, CdCl_2_. All data were derived from at least three biological repeats*.

* *p < 0.05*,

** *p < 0.01*.

Photosynthesis systems and chlorophyll in microalgae are sensitive to environmental stress. The addition of Cd, and N– decreased the chlorophyll content of *E. gracilis* significantly (Table 1), whereas PRM had no significant effect. Chlorophyll content decreased by 55% (*p* < 0.05) and 38% (*p* < 0.001) in cells treated with Cd and N–, respectively.

Similarly, there were obvious differences in the paramylon content of *E. gracilis* under different environmental treatments. PRM did not affect paramylon content; Cd caused a significant increase (171% of the control, *p* < 0.05), whereas N– showed an obvious reduction (66% of the control, *p* < 0.05) (Table 1).

Many environmental stresses could alter the metabolism, cellular membrane structure, and lipids. In this study, significant changes of total lipids were also detected in both Cd and N– treatments, with 53 and 129% increases compared to EM control, respectively. PRM caused no significant change in total lipid content in *E. gracilis*.

### Comparative Metabolomics of *Euglena* Under Different Environmental Stresses

#### General Features of Metabolomics

In this study, the metabolic plasticity of *E. gracilis* under different stresses was elucidated using comparative metabolomic analysis. UHPLC–MS/MS revealed a total of 10,892 negative ion mode (NEG) and 11,003 positive ion mode (POS) peaks. Furthermore, 254 NEG and 583 POS metabolites, respectively, were annotated by the KEGG database (Supplementary Table 1).

**Figure 1:**
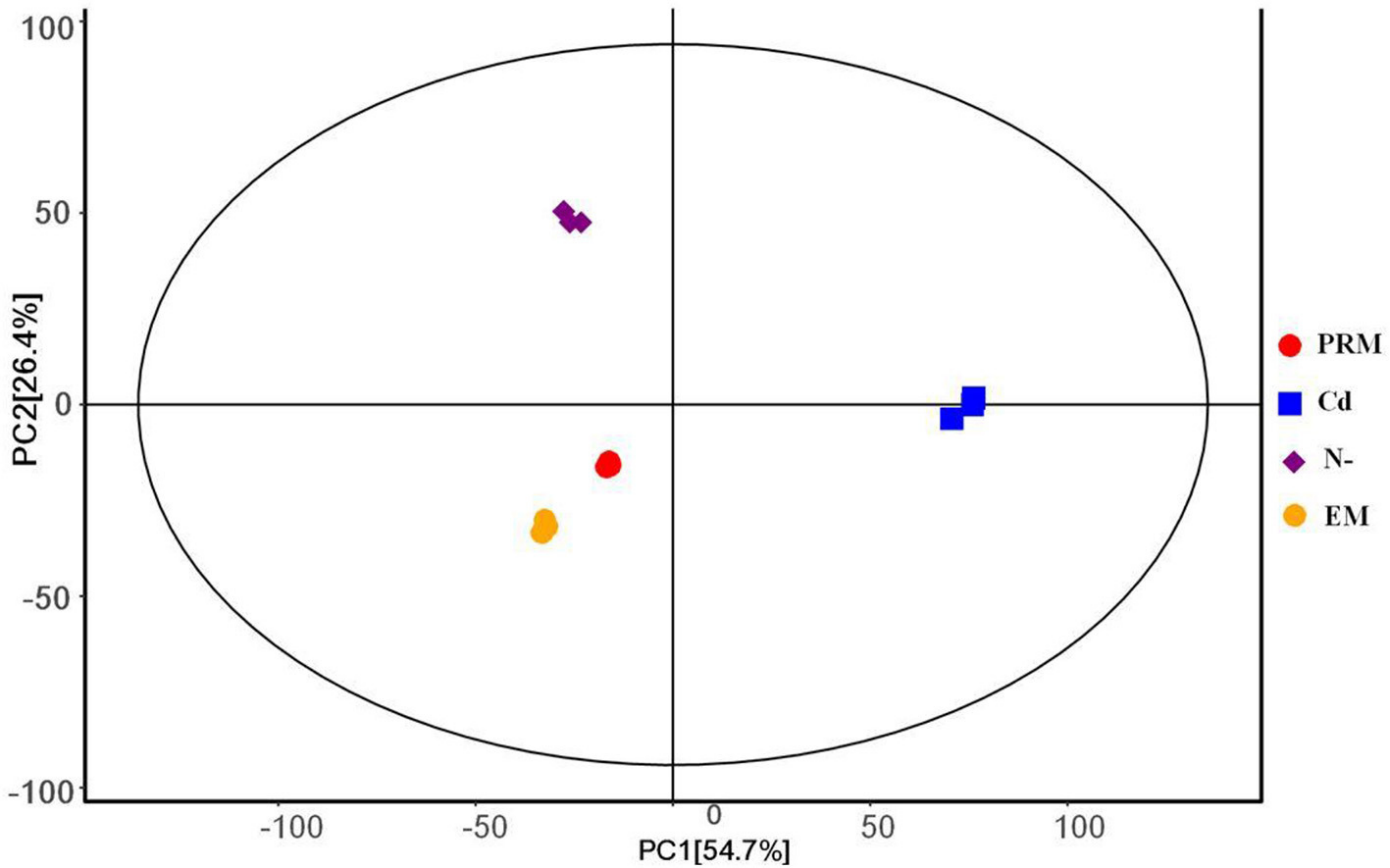
PCA score plot under different treatment groups based on metabolites, each with 3 biological replicates. PRM, paromomycin; Cd, CdCl_2_ addition; N–, nitrogen deprivation; EM, control.

The PCA analysis showed that all four groups, EM, PRM, N–, and Cd, of metabolites could be distinguished (Figure 1), indicating that the metabolic changes to stress were significantly different from each other. Among them, two groups of metabolites (EM and PRM) were relatively closer, indicating that smaller differences had occurred in the metabolic alterations of microalgae cells treated with PRM. Furthermore, the metabolites (N– and Cd) of two groups of microalgae cells were clearly distinguished between each other, and far from EM and PRM groups (Figure 1), suggesting that the metabolic pathways of the microalgae cells changed dramatically under N– and Cd.

**Figure 2:**
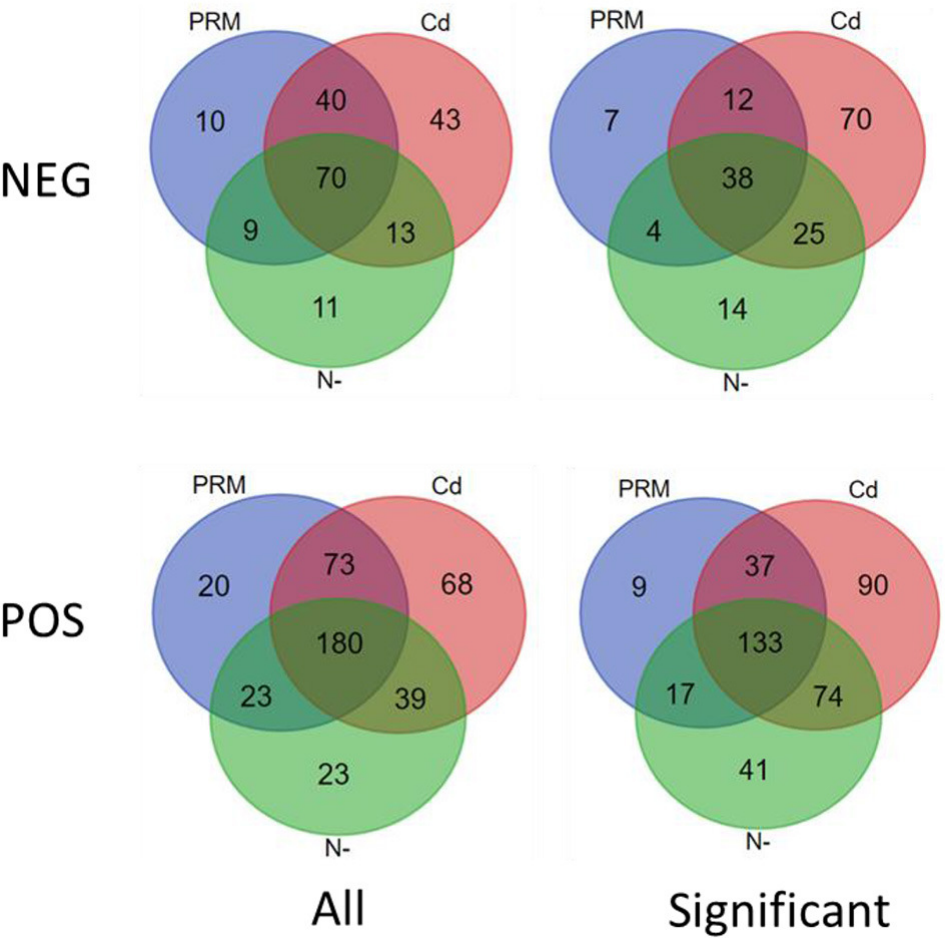
Venn diagram of all (All) and significantly changed (significant) metabolites under stresses (PRM, paromomycin; Cd, CdCl_2_; N–, nitrogen deprivation), under negative (NEG) and positive (POS) ion modes.

According to the criteria of fold change (>2.0, or <0.5, and *p* < 0.05), 401 out of 426 (POS) and 170 of 196 (NEG) candidate metabolites were significantly changed under different treatments (Figure 2).

In total, numbers of significantly decreased metabolites were much more than increased ones in all stress treatment groups, for instance, 177 vs. 19 (PRM) and 313 vs. 21 (Cd) under the POS mode, whereas similar trends were also observed under the NEG mode, for instance, 52 vs. 8 (PRM) and 108 vs. 36 (Cd) (Table 2). These stresses inhibited *Euglena* cell growth by reducing the metabolism levels of cellular activity. Among stresses, Cd seems to cause more dramatic metabolic changes, with the most significantly changed metabolites as total 334 and 166 under POS and NEG mode, respectively, in contrast to N– group (179, 80) and PRM (196, 60) compared to the EM control.

**Table 2:** Summary of metabolomics: differential metabolites under different stresses (PRM, paromomycin; Cd, CdCl2; N–, nitrogen deprivation) and under negative (POS) and positive (NEG) ion modes.

**Mode**	**POS**				**NEG**			
	**Down**	**Up**	**Sum**	**Total *p* < 0.05**	**Down**	**Up**	**Sum**	**Total *p* < 0.05**
PRM	177	19	196	296	52	8	60	129
Cd	313	21	334	360	108	36	144	166
N–	160	19	179	265	72	8	80	103

#### TOP Changed Metabolites Under Stresses

Based on fold changes increased and decreased, TOP 10 metabolites under different stresses were analyzed (Supplementary Table 2). Interestingly, metabolites as organic oxygen compounds, organic acids, and derivatives (mostly amino acids) were deceased, and lipids and lipid-like molecules increased in PRM treatment. Under Cd, TOP changed metabolites include lipids and organoheterocyclic compounds and increased polyketides and benzenoids. While as expected, N– treatment induced significant lipids like PC (16:0/P-16:0; 24:0/14:0), PS (18:1/15:0; 15:0/16:1), DG (20:3), and reduced organic acids (mostly single amino acids as Tyr, Asp, and Arg, and two peptides like Arg-Ser, Thr-Arg, and Arg-Phe).

#### KEGG Pathway Enrichment

**Figure 3:**
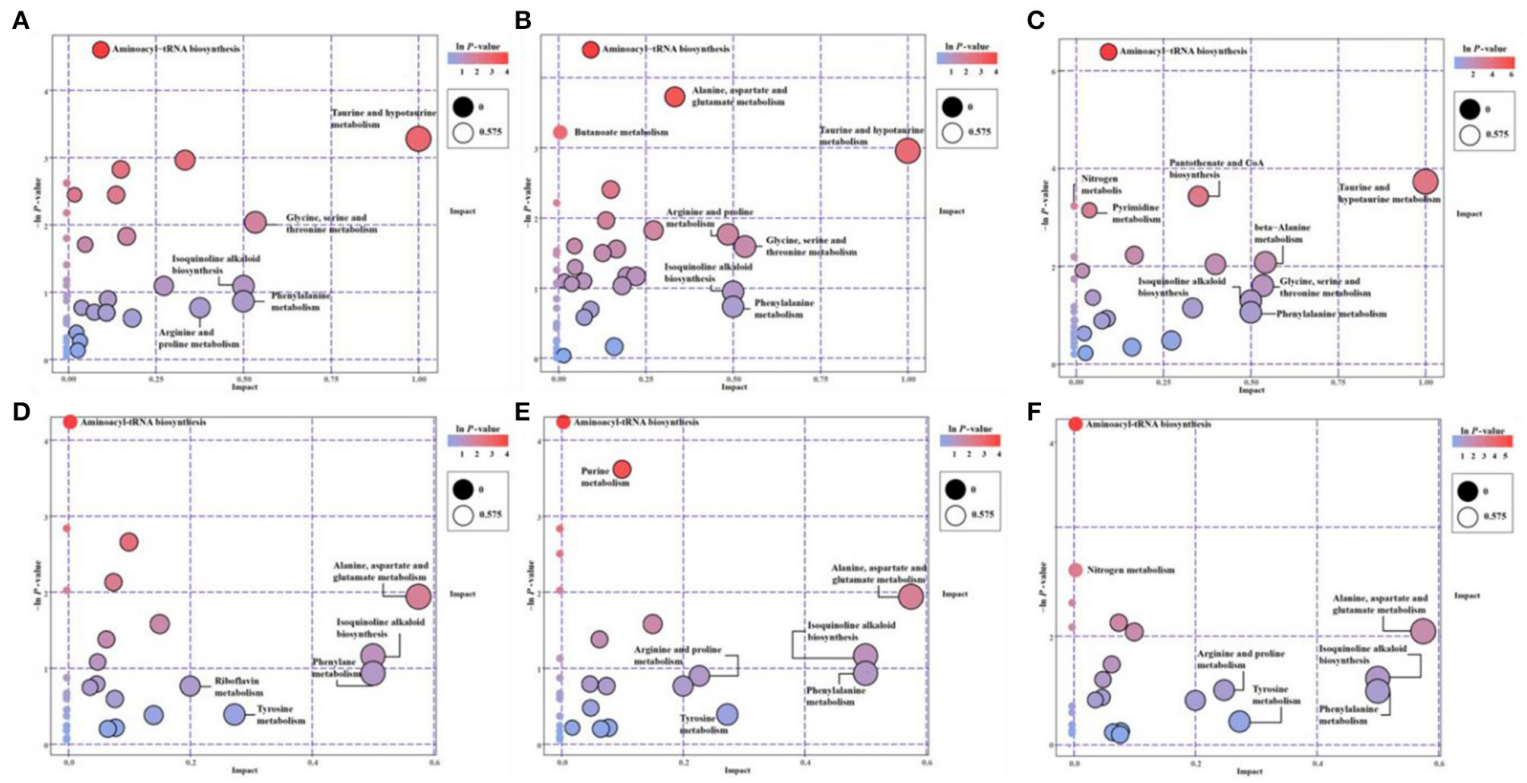
The pathway enrichment and topology analysis of candidate metabolites from *E. gracilis* under different environmental stresses. **(A–C)** NEG modes; **(D–F)** POS modes. **(A,D)** PRM-NEG, PRM-POS; **(B,E)** Cd-NEG, Cd-POS; **(C,F)** N-NEG, N-POS. “Pathway Impact” on the *x*-axis represents the impact of these enriched pathways computed *via* topology analysis. “–log10(*p*)” on the *y*-axis refers to the negative natural logarithmic value of the original *p*-value from a statistical analysis of the pathway difference between the groups of stresses vs. the EM control. The redder the color, the higher the –log10(*p*) and pathway impact value, the more significant the enrichment degree.

The KEGG-enriched metabolome bubble view showed all matched pathways according to the *p-*values from pathway enrichment analysis and the pathway impact values from pathway topology analysis (Figure 3). The candidate metabolites with *p* < 0.05 were enriched by one or several metabolic pathways under three stresses, such as Aminoacyl-tRNA biosynthesis, Purine metabolism, Nitrogen metabolism, “Taurine and hypotaurine metabolism,” Butanoate metabolism, “Alanine, aspartate and glutamate metabolism,” “Pantothenate and coenzyme A (CoA) biosynthesis,” and Pyrimidine metabolism (Figure 3; for more details see Supplementary Table 3 KEGG list only).

Under both POS and NEG modes, the aminoacyl-tRNA biosynthesis showed up under all stresses, which indicated that this metabolic pathway might be a key stress response of *Euglena* cells to different environmental stresses, such as nutrient deprivation, heavy metal, and antibiotics.

Overall, microalgae cell metabolic pathways under PRM conditions were primarily involved in the biosynthesis of Aminoacyl-tRNA, and Taurine and hypotaurine. With higher *p*-values (*p* > 0.05), some highly impacted pathways involved in amino acids, such as “Ala, Asp, and Glu metabolism,” Phe metabolism, Tyrosine metabolism, “Glycine, Ser, and Thr metabolism,” “Arginine and Proline metabolism,” Isoquinoline alkaloid biosynthesis, and Riboflavin metabolism.

Relatively more significantly enriched metabolic pathways were enriched when cells were treated with Cd. In addition to the biosynthesis of aminoacyl-tRNA, 3 more pathways, Purine metabolism, “Ala, Asp, and Glu metabolism,” and Butanoate metabolism, showed up in the Cd treatment. Similarly, all less significant pathways (*p* > 0.05) enriched in PRM were on the Cd pathway list (Figure 3), except for the Riboflavin metabolism.

Compared with PRM and Cd, N— treatment enriched the most significant metabolic pathways, including Aminoacyl-tRNA biosynthesis, “Taurine and hypotaurine metabolism,” “Pantothenate and CoA biosynthesis,” Nitrogen metabolism, and Pyrimidine metabolism. The late three pathways were N– group-specific, and nitrogen metabolism as the well-known pathways changed when nitrogen nutrient was absent.

#### Network Analyses of Metabolomics

Based on the metabolomic KEGG and network analyses, we also obtained groups of metabolic pathways and modules that play core roles in the metabolic networking (Table 3 network plot). Several pathways and modules were plotted in all stress groups simultaneously, including Purine metabolism (module cvr00230), “Ala, Asp, and Glu metabolism” (cvr00250), Carbon fixation in photosynthetic organisms (cvr00710), Guanine ribonucleotide biosynthesis (M00050), Glycerophospholipid metabolism (cvr00564), and PRPP biosynthesis (M00005). M00005 is a module on KEGG, The KEGG MODULE database consists of KEGG modules identified by M numbers and KEGG reaction modules identified by RM numbers, which are manually defined functional units of gene sets and reaction sets, respectively. KEGG modules are further divided into pathway modules and signature modules as shown https://www.genome.jp/entry/M00005, and the PRPP: 5-Phosphoribosyl-1-Pyrophosphate.

**Table 3:** Network plots of significantly enriched pathways and modules under stresses.

**PRM**	**Cd**	**N–**	**Pathways and modules description**
**NEG**			
cvr00230	cvr00230	cvr00230	Purine metabolism
cvr00250	cvr00250	cvr00250	Alanine, aspartate, and glutamate metabolism
cvr00710	cvr00710	cvr00710	Carbon fixation in photosynthetic organisms
M00050	M00050	M00050	Guanine ribonucleotide biosynthesis IMP
M00168	M00168		CAM (Crassulacean acid metabolism), dark
M00169	M00169		CAM (Crassulacean acid metabolism), light
M00172	M00172		C4-dicarboxylic acid cycle, NADP—malic enzyme
M00005			PRPP biosynthesis, ribose 5P = > PRPP
	cvr00430		Taurine and hypotaurine metabolism
	M00028	M00028	Ornithine biosynthesis, glutamate = > ornithin
		cvr04075	Plant hormone signal transduction
		cvr00590	Arachidonic acid metabolism
		M00052	Pyrimidine ribonucleotide biosynthesis
**POS**
cvr00230	cvr00230	cvr00230	Purine metabolism
cvr00564	cvr00564	cvr00564	Glycerophospholipid metabolism
M00005	M00005	M00005	PRPP biosynthesis, ribose 5P = > PRPP
	M00050	M00050	Guanine ribonucleotide biosynthesis IMP = > GDP
cvr00591	cvr00591		Linoleic acid metabolism
M00092	M00092		Phosphatidylethanolamine (PE) biosynthesis
M00094			Ceramide biosynthesis
M00099			Sphingosine biosynthesis
	cvr00071		Fatty acid degradation
	cvr00232		Caffeine metabolism

On both PRM and Cd network lists, we found Crassulacean acid metabolism (CAM), dark (M00168) and light (M00169); C4-dicarboxylic acid cycle, nicotinamide adenine dinucleotide phosphate (NADP)-malic enzyme (M00172), Linoleic acid metabolism (cvr00591), and Phosphatidylethanolamine (PE) biosynthesis (M00092). However, on both Cd and N– lists, only two networks were detected as Guanine ribonucleotide biosynthesis (M00050) and Ornithine biosynthesis (M00028).

Several specific networks were only detected in one individual stress. For examples, Ceramide biosynthesis (M00094) and Sphingosine biosynthesis (M00090) in the PRM group; Fatty acid degradation (cvr00071) and caffeine metabolism (cvr00232) in the Cd group; Plant hormone signal transduction (cvr04075), Arachidonic acid (ARA) metabolism (cvr00590), and Pyrimidine ribonucleotide biosynthesis (M00052) under N– group.

## Discussion

*Euglena* is highly adaptable to changing environments, some adverse stresses like UVB (Takahashi et al., 2006), heavy metals (Moreno-Sánchez et al., 2017), and antibiotics (Ebringer, 1964). In this study, microalgal cells showed tolerance to antibiotics, PRM, without significant cell growth inhibition, chlorophyll reduction, and paramylon and lipid alterations. The inhibition of chlorophyll formation in *Euglena* by antibiotics was reported previously (Linnane and Stewart, 1967). This may be caused by different antibiotics and different tolerant concentrations of *E. gracilis* cells. In the Cr(VI) treatments, a reduction of chlorophyll was observed, the ratio of proteins to paramylon content was augmented, and total lipid content was increased (Rocchetta et al., 2012). In contrast, the other two stresses, N– and Cd, showed obvious effects on above-mentioned physiological parameters and major macromolecules, such as polysaccharide (paramylon) and lipids.

This is the first time to apply global metabolomics to study the cellular response of *E. gracilis* to environmental stresses, such as antibiotics, heavy metals, and nutrient deprivation. Many metabolites and metabolic pathways, instead of standard physiological parameters, and macromolecules, were detected significantly altered by these stresses and shed light on the mechanisms of tolerance or toxic responses in *E. gracilis*. PRM did not cause much change of pigment, carbohydrates, and lipids in *E. gracilis* cells. However, based on metabolomics, hundreds of metabolites and several important metabolic pathways were detected significantly disturbed by PRM, indicating the sensitivity of metabolomics when studying the microalgal cellular response to changing environments. Metabolomics easily differentiates the groups of control, PRM, N–, and Cd treatments, even between control and PRM, by which cell growth, chlorophyll content, paramylon, and lipid changes could not distinguish significantly.

In this study, we detected paramylon degradation and lipids accumulation under N–. In most microalgae, lipids were induced to high accumulation under nitrogen starvation (Chen et al., 2017). There is a report showing induction of carbohydrates and no lipid accumulation with no nitrogen source in *Euglena* with NH_4_Cl as the only nitrogen source (Coleman et al., 1988). However, there is a major difference compared to the reference above since we added urea and NH_4_Cl as nitrogen sources. Thus, N– in this study means both NH_4_Cl and urea depletion and may cause differential accumulation (reduction) of carbohydrates and lipids in our study.

Based on hundreds of changed metabolites, significantly increased or decreased TOP 10 metabolites may suggest the major changes under different treatments. Even with no significantly changed lipids and paramylon under the PRM treatment, amino acids were found to decrease while lipids and lipid-like molecules increase. PRM is a broad-spectrum aminoglycoside antibiotic produced by *Streptomyces rimosus* var. *paromomycinus* (Davidson et al., 2009). The *in vitro* and *in vivo* antibacterial action of PRM closely parallels that of neomycin. PRM bind specifically to the RNA oligonucleotide at the A site of bacterial 30S ribosomes thereby causing misreading and premature termination of translation and leading to inhibition of protein synthesis followed by cell death (PubChem CID 165580). As reported, some antibiotics, such as chloramphenicol and other antibiotics that specifically inhibit the synthesis of proteins in mitochondria and bacteria, would also selectively inhibit protein synthesis by chloroplast (Linnane and Stewart, 1967). PRM did not reduce chlorophyll biosynthesis in *E. gracilis* cells at the concentration of 25 μg/ml, suggesting its inhibition pathways may not involve the synthesis of chloroplast proteins. Cd treatment in this study resulted in chlorophyll degradation, paramylon, and lipid accumulation. Under Cd treatment, metal-causing reactive oxygen species (ROS) changed the lipids and organoheterocyclic compounds, and N– increased lipids and decreased protein contents in *Euglena* cells. These TOP changed metabolites showed similar patterns as previous reports (Moreno-Sánchez et al., 2017; Tossavainen et al., 2019).

The KEGG enrichment provides metabolic pathways through which cells are regulated under specific treatments. The only common pathway enriched in all treatments is Aminoacyl-tRNA biosynthesis, suggesting this may be the common/universal stress responding pathway in *Euglena*. Aminoacyl-tRNA species are prevalent amino acid donors in the cell due to their critical role as substrates for ribosomal protein biosynthesis. It has become clear that aminoacyl-tRNAs are also utilized in an ever-growing number of other non-ribosomal biosynthetic pathways, particularly in bacteria, although some pathways also exist in eukaryotes (Shepherd and Ibba, 2013). Much has been uncovered in recent years about many novel functions, disease connection, and inter pathway connection of tRNA synthetases (Park et al., 2008; Zhou et al., 2020). PRM might inhibit protein synthesis by binding specifically to the RNA oligonucleotide at the site of bacterial, or microalgal mitochondrial and 30S ribosomes of chloroplast, and thus alter aminoacyl-tRNA biosynthesis. Nitrogen deprivation involves the degradation of carbohydrates, paramylon in *E. gracilis*, and constructive proteins, and accumulation of membrane lipids (Richter et al., 2015), and at the same time, specific enzymes/macromolecules have to be transformed or *de novo* synthesized *via* aminoacyl-tRNA biosynthesis pathway. Similarly, heavy metals are created ROS through lipid peroxidation and DNA damage (Rodriguez-Zavala et al., 2007; Khatiwada et al., 2020). Then the cells are induced to produce antioxidant chemicals and enzymes, such as superoxide dismutase and DNA repair proteins from protein biosynthesis with aminoacyl-tRNA biosynthesis pathway. However, whether the aminoacyl-tRNA biosynthesis pathway could be a novel biomarker for environmental stresses has yet to be worked out in the future with more investigations.

There are more pathways enriched under individual stresses according to our global metabolomics, reflecting different metabolic responses by different stresses.

In Cd treatment, “Purine metabolism,” “Alanine, aspartate, and glutamate metabolism,” and “Butanoate metabolism” pathways were detected significantly changed. Purine nucleotides are essential for many biochemical processes like energy transfer, metabolic regulation, and synthesis of DNA and RNA (Watanabe et al., 2014). Alanine, aspartate, and glutamate are derived from intermediates of central metabolism, mostly the citric acid cycle, in one or two steps. While the pathways are short, the importance and complexity of the functions of these amino acids befit their proximity to central metabolism (Limami et al., 2008). Butanoate metabolism describes the metabolic fate of several short-chain fatty acids or short-chain alcohols. Many of these molecules are eventually used in the production of ketone bodies, the creation of short-chain lipids, or as precursors to the citrate cycle (pubchem.ncbi.nlm.nih.gov/pathway/PathBank:SMP0000073). Cd degraded chlorophyll and reduced the activity of photosynthesis of microalgal cells; however, short-term (6 days) Cd treatment also increased the contents of paramylon and lipids (Mendoza-Cozatl et al., 2002; Einicker-Lamas et al., 2003). Thus, we speculated that the mutual transformation of three major macromolecules, proteins, carbohydrates, and lipids may respond to heavy metals. In Cd treatment, decreasing amino acid contents and significantly enriched protein synthesis pathways with enhanced carbohydrates and lipid production were detected, indicating the mutual transformation of proteins into paramylon and lipids in *E. gracilis*.

When microalgal cells were treated with PRM, the “Taurine and hypotaurine metabolism” pathway was also enriched. Taurine, an active substance that regulates the normal physiological activities of cells, is associated with maintaining the osmotic pressure balance in cells, plus strengthening cell membranes and enhancing antioxidant capacity (Tevatia et al., 2015, 2019). Taurine is synthesized in large amounts by animals; however, plants, green microalgae, and fungi could only synthesize minute amounts (Tevatia et al., 2019). *E. gracilis* enriched this metabolic pathway where the metabolite was located under PRM treatment. Thus, our results indicated that the metabolic pathway played an important role in regulating antibiotics tolerance of *E. gracilis*, as it stabilized the cell membrane and maintained cellular functions.

Nitrogen deprivation also enriched the taurine and hypotaurine compounds, suggesting this pathway may also involve in nutrient deprivation stress perhaps just by using taurine and its relative amino acid compounds as alternative nitrogen resources for biosynthesis of new enzymes for maintaining basic cell survival. Under N– treatment, KEGG pathway enrichment also collected other pathways such as “Nitrogen metabolism,” “Pantothenate and CoA biosynthesis,” and “Pyrimidine metabolism.”

Nitrogen makes up only a small fraction of plant and microalgal dry weight, but N compounds are extremely important physiologically. For instance, structural and storage proteins, enzymes, amino acids and amides, nucleic acids, and plant hormones, all contain N. The substituted purine and pyrimidine bases that constitute nucleic acids, nucleotides, and nicotinamide nucleotides also contain N (Pallardy, 2008). Transcriptome analysis revealed that genes involved in metabolism, plant hormone signal transduction (e.g., abscisic acid, auxin, and jasmonate), transporter activity, and oxidative stress responses were rapidly regulated by N– (based on our unpublished transcriptomic data in N– *Euglena*).

Pantothenate is vitamin B5 and is the key precursor for the biosynthesis of CoA, a universal and essential cofactor involved in a myriad of metabolic reactions, including the synthesis of phospholipids, the synthesis and degradation of fatty acids, and the operation of the tricarboxylic acid cycle (Leonardi and Jackowski, 2007). Pyrimidines metabolism pathway suggests the cells use the nitrogen from nucleic acids under N–. Although both pyrimidines and purines are components in nucleic acids, they are made in different ways. Likewise, products of pyrimidine degradation are more water-soluble than are the products of purine degradation (Wan et al., 2019).

In summary, the KEGG pathway enrichment showed that both common and specific metabolic pathways were employed to respond against different environmental stresses by *E. gracilis* cells.

Based on network plot analyses, we also targeted some specific metabolic pathways and modules under different stresses, especially on photosynthesis and carbon fixation, ROS, membrane lipids, and amino acid metabolisms.

Photosynthesis is a global sensor of environmental stress in green plants, microalgae, and cyanobacteria (Biswal et al., 2011). Photosynthesis and photosynthetic pigments in *E. gracilis* were reported as sensitive endpoints for the toxicity evaluation of liquid detergents (Azizullah et al., 2014). Carbon fixation is the process by which inorganic carbon is added to an organic molecule, and it occurs during the light-independent reaction of photosynthesis with the help of chlorophyll. This pathway is showed as a core responsive pathway in all three stresses in metabolic networking analyses, indicating photosynthesis and carbon fixation involves in the alternations caused by antibiotics, heavy metals, and N–. Cd and N– treatments significantly reduced chlorophyll contents with lower photosynthetic activity, whereas PRM did not decrease chlorophyll significantly. Further experiments with the photosynthetic activity will clarify this observation.

It is very interesting to locate the CAM pathway in PRM and Cd groups. CAM (also known as CAM photosynthesis) is a carbon fixation pathway that evolved in some plants as an adaptation to arid conditions. In CAM, the carboxylation (C3 + C1) occurs at night, and the (C4 – C3) decarboxylation occurs during the day (Libik-Konieczny et al., 2019; Gilman and Edwards, 2020). The C3 + C1 mechanism has been found in the green Ulvophyceae benthic macroalga *Udotea flabellum* and the planktonic diatom *Thalassiosira weissflogii* (Cheng et al., 2019). The photosynthetic metabolism (C3, C4, or CAM) seems to affect plant sensitivity to environmental stresses. CAM is a metabolic strategy allowing plants to maintain photosynthesis under stress conditions (Libik-Konieczny et al., 2019). Although N– also showed a significant reduction of chlorophyll contents as Cd treatment; however, only PRM and Cd triggered CAM metabolism changes. Heterotrophic CO_2_ fixation by *E. gracilis* was reported in Levedahl (1966). A previous study indicated that *E. gracilis* can remove Cd under anaerobic conditions, which might be advantageous for heavy metal removal in sediment from polluted water bodies or bioreactors, where the O_2_ concentration is particularly low (Geovanni Santiago-Martinez et al., 2015). Perhaps under PRM and Cd treatments, the damage of photosynthesis might be compensated partially by CAM metabolism, which needs further verification shortly.

Finally, *Euglena* appears to exemplify a strategy for survival and adaptation to various environmental conditions during the evolutionary process of euglenoids (Geovanni Santiago-Martinez et al., 2015; Ishikawa et al., 2017). Among various environmental stresses, heavy metal accumulation and resistance in *Euglena* are widely studied. Under Cd treatment, mitochondria and chloroplasts were altered in shape, and thylakoid arrangement was changed, and osmiophilic plastoglobuli was increased (Duret et al., 1986). Cd causes cellular damage to *Euglena*, including DNA strand breaks and intracellular membrane damage, as a result of reactive oxygen stress (Watanabe and Suzuki, 2002). Enhanced alternative oxidase and antioxidant enzymes under Cd stress in *Euglena* were reported (Castro-Guerrero et al., 2008). The data suggested that Cd was compartmentalized into chloroplasts in a process that may involve the transport of free Cd and the participation of thiol-peptides in *E gracilis* (Mendoza-Cozatl et al., 2002). The chloroplasts appear to have a major role in the tolerance and accumulation of Cd in *E. gracilis* (Khatiwada et al., 2020). The variety of biochemical mechanisms evolved in *E. gracilis* to resist, accumulate, and remove heavy metals from the environment are summarized as follows: adsorption to the external cell pellicle, intracellular binding by glutathione and glutathione polymers, and their further compartmentalization as heavy metal complexes into chloroplasts and mitochondria, polyphosphate biosynthesis, and secretion of organic acids (Moreno-Sánchez et al., 2017).

Alternation of membrane lipids and PUFA pathways was also reported in all stresses. Lipid metabolism is crucial for the survival and propagation of the species since lipids are an essential cellular component for maintaining homeostasis in the presence of environmental stressors. Reactions in glycerolipid and glycerophospholipid metabolism occur at the membrane, which plays an important role in signaling and response to environmental stress. PE is one of the most abundant membrane phospholipids, in which metabolism was significantly changed under PRM and Cd. In both prokaryotes and eukaryotes, PE plays important role in various membrane functions. It is suggested that the synthesis of these membrane lipids was beneficial to increase the peristalsis and swimming ability of *E. gracilis*, which protected the integrity of the cell, improving the viability of the cell under these stresses.

Under the PRM treatment, ceramide biosynthesis and sphingosine metabolism were also disturbed. Ceramide biosynthesis begins in the endoplasmic reticulum with the condensation of palmitoyl-CoA and serine, catalyzed by the multimeric enzyme serine palmitoyltransferase to produce 3-ketosphinganine (Chaurasia and Summers, 2015). Sphingosine is a long-chain unsaturated amino alcohol C18H37O2N that is found especially in cell membranes and is a primary constituent of sphingolipids. Sphingosine itself increases the permeability of phospholipid membranes. With no cell walls in *E. gracilis* unlike other microalgae, the external cell pellicle and the cell membrane are the first front-line against the changing aquatic environment. Thus, distortions and alterations of membrane lipids of *Euglena* have to be sensitive and elastic in the environment.

As the major compound of membrane lipids, PUFAs are also very sensitive to environmental stresses at qualitative and quantitative levels. Halter et al. (2012) found that under adverse conditions, such as the presence of heavy metal arsenic, the content of unsaturated fatty acids in the cell membrane had significantly increased. In this study, linoleic acid (LA) metabolism was highlighted in groups of PRM and Cd (Halter et al., 2012). LA [18:2 (*n*−6)] is an omega-6 fatty acid and helps stimulate skin and hair growth, maintain bone health, regulate metabolism, and to maintain the reproductive system (Lee et al., 2018). Under abiotic stress by salt or ultraviolet light, the production of lutein and PUFAs were observed by the acidophilic eukaryotic microalga *Coccomyxa onubensis* (Bermejo et al., 2018). Thus, increasing the fluidity and hydrophobicity of the cell membrane by increasing PUFA allowed *E. gracilis* adaptability in the surrounding environments, such as PRM and Cd. Similar PUFA accumulation was also reported in freshwater microalgae *Chlamydomonas mexicana* and *Scenedesmus obliquus* grown under salt stress (Salama et al., 2013). Furthermore, it was determined that there is a relationship between the effects of Cd-induced oxidative stress on PUFAs and their degradation products and is also reflected on the increase in tolerance to Cd toxicity in plant cells (Pavlík et al., 2017). Specifically to N–, ARA metabolism reacted significantly. ARA is an integral constituent of the biological cell membrane, conferring it with fluidity and flexibility, so necessary for the function of all cells. Free ARA modulates the function of ion channels, several receptors, and enzymes *via* activation as well as inhibition (Tallima and El Ridi, 2018). Similarly, the green oleaginous microalga *Lobosphaera incisa* accumulates triacylglycerols enriched in the long-chain PUFA, such as ARA under nitrogen deprivation. This is consistent with the enhanced lipid accumulation under N– stress in *E. gracilis*.

## Conclusions

This study demonstrated that metabolomics is sensitive and accurate enough to elucidate cellular responses of *E. gracilis* to different environmental stresses, even with no significantly changed physiological parameters, such as cell numbers, pigments, paramylon, and lipids under PRM treatment. Based on global metabolomics, a common metabolic pathway responding to multiple environmental stresses, aminoacyl-tRNA biosynthesis, was firstly targeted in *E. gracilis*. The other shared metabolic pathways and modules under selected stress include Purine metabolism, carbon fixation, Ala, Asp, and Glu metabolism, Glycerophospholipid metabolism, and PRPP biosynthesis. We observed the well-known changed metabolic pathways involved in stresses, like ROS in heavy metals, N metabolism during N deprivation, and protein biosynthesis inhibition with PRM. Some unique modules were individually detected under different stresses, like ceramide and sphingosine biosynthesis under PRM, ARA metabolism in N–, and fatty acid degradation and caffeine metabolism with Cd treatment. Under stresses, without cell walls, membrane lipids and PUFA in *E. gracilis* cells were altered to resist, acclimatize to, and remove the adverse factors like Cd and reactive oxidative species. All data suggest that the mutual interaction and transformation of macromolecular metabolites as proteins, pigments, lipids, and paramylon are regulated delicately to resist, tolerate, and/or remove these environmental stresses.

## Data Availability Statement

The data presented in the study were deposited in the MetaboLights repository, with the accession number MTBLS2878.

## Author Contributions

JW and ZH contributed to the conception and design of the study. JH, CL, MD, and XZ conducted the experiments. JH, CL, AL, and XZ involved in the statistical analysis. JW and JH wrote the first draft of the manuscript. AL and JW wrote the sections of the manuscript. JW and CL contributed to manuscript revision. All authors read and approved the submitted version.

## Conflict of Interest

The authors declare that the research was conducted in the absence of any commercial or financial relationships that could be construed as a potential conflict of interest.

## Footnotes

^1^https://www.metaboanalyst.ca/MetaboAnalyst/home.xhtm.

## Abbreviations

ARA: arachidonic acid
CAM: Crassulacean acid metabolism
CoA: coenzyme A
*E. gracilis*: *Eugleaa gracilis*
KEGG: Kyoto Encyclopedia of Genes and Genomes
OPLS-DA: orthogonal projections to latent structures discriminant analysis
PCA: principal component analysis
PE: phosphatidylethanolamine
PRM: paromomycin
PUFAs: polyunsaturated fatty acids
ROS: reactive oxygen species
VIP: variable importance in projection.
