# Supplementary material for: Metabolic Responses of a Model Green Microalga Euglena gracilis to Different Environmental Stresses
Source: Front Bioeng Biotechnol. 2021 Jul 20;9:662655. doi: 10.3389/fbioe.2021.662655 (PMC8329484; doi:10.3389/fbioe.2021.662655)
Supplement: Supplementary file 3 [file Table_3.pdf]

	POS				NEG			
	**Pathway**	**Total**	**Hits**	**Raw p**	**Pathway**	**Total**	**Hits**	**Raw p**
**PRM**	Aminoacyl-tRNA biosynthesis	67	9	0.01652	Aminoacyl-tRNA biosynthesis	67	10	0.010068
					Taurine and hypotaurine metabolism	5	2	0.037775
**Cd**	Aminoacyl-tRNA biosynthesis	67	9	0.01652	Aminoacyl-tRNA biosynthesis	67	11	0.012337
	Purine metabolism	61	8	0.026919	Alanine, aspartate and glutamate metabolism	22	5	0.024123
					Butanoate metabolism	18	4	0.046332
**N-**	Aminoacyl-tRNA biosynthesis	67	10	0.0033136	Aminoacyl-tRNA biosynthesis	67	10	0.0017023
	Nitrogen metabolism	15	3	0.048602	Taurine and hypotaurine metabolism	5	2	0.024079
					Pantothenate and CoA biosynthesis	14	3	0.032429
					Nitrogen metabolism	15	3	0.039058
					Pyrimidine metabolism	38	5	0.043252
